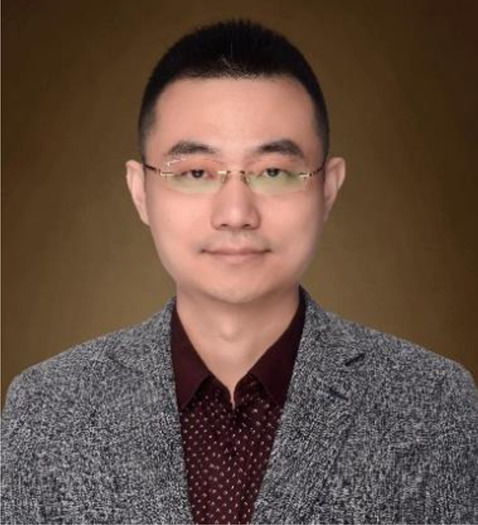# Light People: Professor Dayong Jin

**DOI:** 10.1038/s41377-021-00673-9

**Published:** 2021-11-27

**Authors:** Ying Zhang

**Affiliations:** Light Publishing Group, Changchun Institute of Optics, Fine Mechanics and Physics, Chinese Academy of Sciences, 3888 Dong Nan Hu Road, Changchun, 130033 China

## Abstract

He pioneered a new family of nanoscopic probes that can up-convert infrared photons into intense visible light, and won the Australian Museum Eureka Prize for Interdisciplinary Scientific Research in 2015. He created new kinds of microscopes that allow us to watch molecules at work inside living cells, and won the Australian Prime Minister’s Prize for Science Malcolm McIntosh Prize for Physical Scientist of the Year 2017. The Australian newspaper identified him among 100 “rock stars of Australia’s new economy” as the Knowledge Nation 100. This year, at his age of 42, he won the Australian Laureate Fellowship and was elected to the fellowship of Australian Academy of Technology and Engineering. This is Dayong Jin, a Distinguished Professor at the University of Technology Sydney and a Chair Professor at Southern University of Science and Technology, as well as the editorial manager in Sydney office and the perspective column editor of Light: Science & Applications (LSA). Light People is a featured column of high-end interviews with outstanding scientists. On this issue, it is our great honor to invite Professor Dayong Jin to provide his perspectives on his work, end-user driven research, student mentoring and team building philosophy. In the following, let’s take a closer look at the research life of Professor Dayong Jin, and appreciate his style and the story behind his success.

**Biography:** Dayong Jin is a Distinguished Professor at the University of Technology Sydney (UTS) since 2017 and a Chair Professor at Southern University of Science and Technology (SUSTech) since 2019. Prof Jin obtained his PhD from Macquarie University in 2007. At Macquarie, he was promoted to Lecturer in 2010, Senior Lecturer in 2013, Associate Professor in 2014, and Professor in 2015.

At UTS, as the director, he established the Australian Industry Transformation Research Hub for Integrated Devices for End-user Analysis at Low Levels (ARC IDEAL Hub), the Department of Industry, Science, Energy and Resources’ Australia-China Joint Research Centre for Point of Care Testing (DISER POCT), the UTS-SUSTech Joint Research Centre for Biomedical Materials & Devices, and three major programs underpin the UTS Institute for Biomedical Materials & Devices (IBMD), to transform advances in photonics and materials into disruptive biotechnologies.

**Figure:**
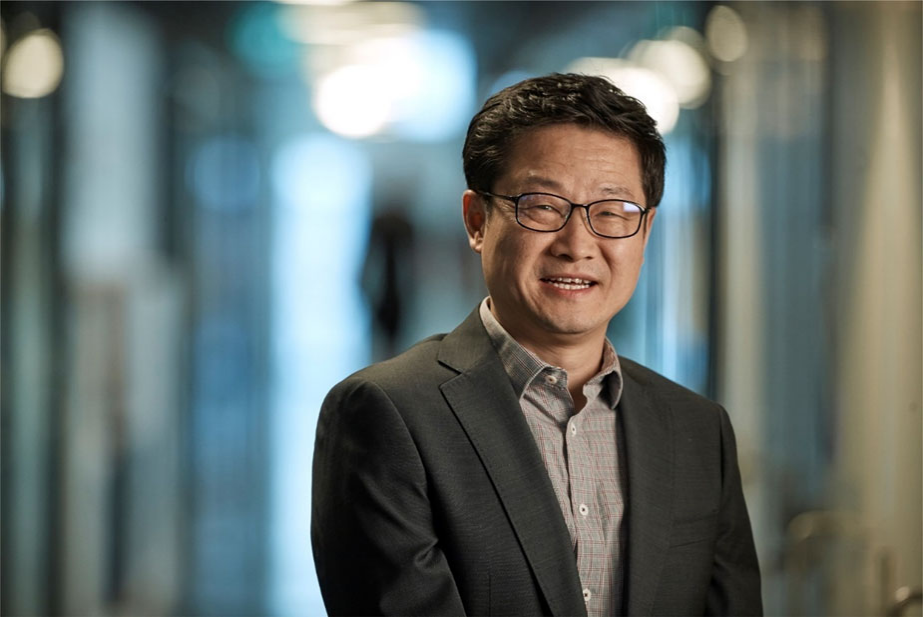

His research has been in the physical, engineering and interdisciplinary sciences, with expertise covering biomedical optics, nanotechnology, luminescent materials, microscopy, diagnostics, and microfluidics devices.

Prof Jin is the winner of the Australian Museum Eureka Prize for Interdisciplinary Scientific Research in 2015, the Australian Academy of Science John Booker Medallist in 2017, and the Prime Minister’s Prize for Physical Scientist of the Year 2017. In the year of 2021, Professor Jin won the Australian Laureate Fellowship and was elected to the fellowship of Australian Academy of Technology and Engineering.

**Q1: First of all, congratulations on your being awarded the Australian Laureate Fellowship and being elected to the fellowship of Australian Academy of Technology and Engineering. Would you like to tell us more about your research in relation to important achievements?**

Indeed, these are the Australian highest recognitions of our works from basic research to applied science and engineering, and our significant contributions to the Australian research landscape by building core resources and consortiums in imaging, cytometry and point-of-care diagnostics, and leading international research programs that are transforming advances in photonics and materials into biotechnology.

The Laureate fellowship in Australia supports the world leaders to play a sustained leadership and significant mentoring role in building Australia’s internationally competitive research capacity. Through the Fellowship, I will build a Laureate team and grow our strengths in super-resolution imaging and single molecular sensing, and develop technologies to map the structures and functions of subcellular compartments and single molecules, so that biologists can then drop-down to get a ‘street view’ of the molecular traffic within cells to decode the complexities of life at work and the early signs of diseases.

**Figure 1:**
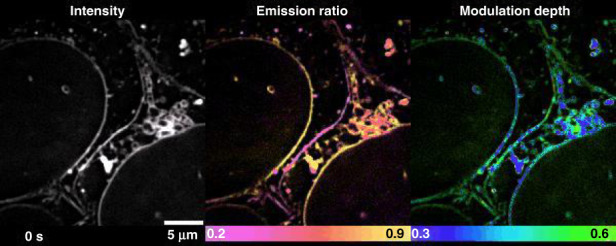
**Time-lapse high-dimensional super-resolution imaging of the late-stage division of two U2-OS cells. Super-resolution “street view” microscopy hits the SPOT**^1^

The fellowship of the Australian Academy of Technology and Engineering recognizes our efforts in the translational research from basic science to new technology developments. Over the past decade, our profile is increasingly known among Australian biotechnology SMEs as a source of advice on photonics and biomedical imaging devices, nanotechnology and point of care testing technologies. We have developed disruptive technologies for Australian biotechnology, diagnostics, veterinary, agribusiness, and manufacturing firms. Through the fellowship, I will work with leaders in academia, industry and government, to provide innovative solutions across a range of areas in addressing issues of critical importance for Australia. In particular, we will focus on the endusers-driven research and impact-driven research to help the Australian community and international countries to recover from this current pandemic.

**Figure 2:**
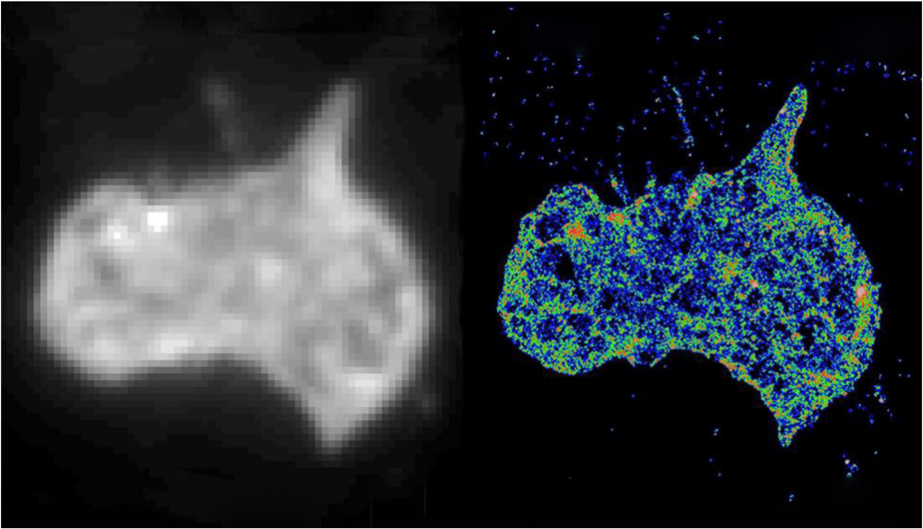
**Joining the dots for ‘street view’ of single human cells. A single platelet cell under a normal microscope, left, and viewed with super-resolution imaging**

**Q2: In 2003, you went to Macquarie University to pursue your PhD. Your PhD supervisor Professor Jim Piper is a renowned laser physicist. At that time, you chose an engineering science topic to develop a new flow cytometer. You then established the advanced cytometry laboratory. Would you like to tell us what made you interested in the research and development of biomedical instruments?**

First of all, I would like to acknowledge my PhD supervisor Professor Jim Piper. He’s a great supervisor, an excellent engineering scientist, and a highly respected laser physicist, who always encourages me to direct my fundamental research towards practical outcomes. Under his supervision, several generations of his students become national and international leaders, including Dr Larry Marshall, the current CEO of the Commonwealth Scientific and Industrial Research Organisation.

**Figure 3:**
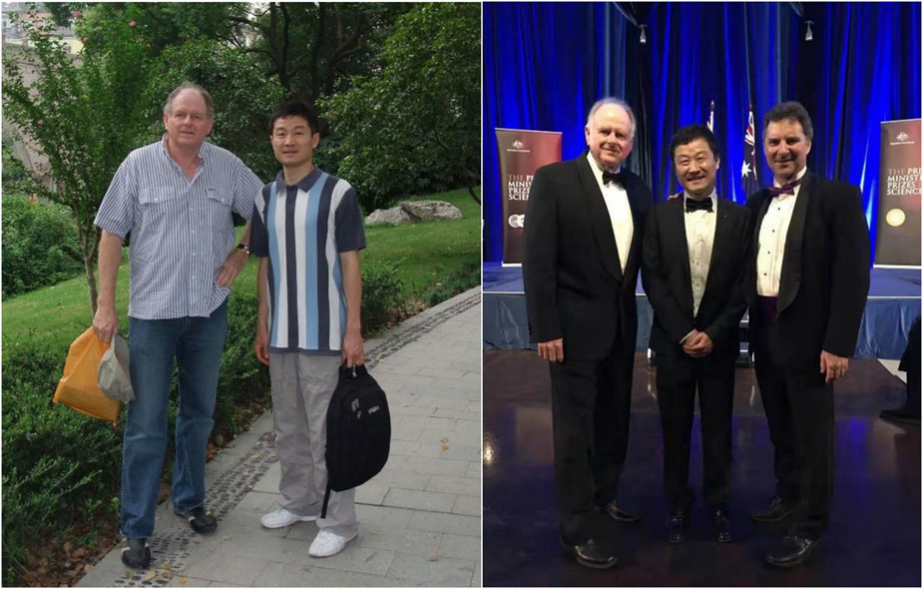
**Photo with my PhD mentor Prof Jim Piper in 2008. Photo with Professor Jim Piper (Left) and Dr Larry Marshall (Right) on the 2017 Prime Minister Prize for Science ceremony**

When I started my PhD at Macquarie University in 2003, Professor Piper suggested two topic areas, to build more efficient lasers, or to develop biomedical devices for cell analysis. From my early trainings in physics and optical engineering, I was deeply inspired by translating laser science into biomedical device applications, and I chose to conduct research in developing flow cytometry. This allows me to learn the multidisciplinary skills, not only in optics, but also in microfluidics, electronics, data acquisition and programming, fluorescent probes, and cell biology.

I completed the three years of my PhD research with an an international patent, which is a very valuable experience to my science career. You may find that most of the research contributions, later on I made in chemistry, material sciences and cell biology, directly benefit from my PhD training as a technology developer and instrumentation builder.

**Q3: In 2015, following your success in Macquarie University, you joined the University of Technology Sydney. In only five years, you have successfully established the Institute for Biomedical Materials & Devices, the Australian Industry Transformation Research Hub for Integrated Devices for End-user Analysis at Low Levels, and the Australia-China Joint Research Centre for Point of Care Testing. Could you please share with us your successful experience in building these multidisciplinary research entities? Have you encountered any challenges? How did you overcome them?**

It is all because of my “ambition”! UTS Vice-Chancellor and President, Professor Attila Brungs, describes me as an ambitious “builder”, which I agree, as I always look for new potentials, make designs and plans, and take the challenges to build new projects. After building the advanced cytometry lab and the node of ARC Centre of Excellence for Nanoscale Biophotonics at Macquarie University, I decided to give myself a new challenge.

My move to UTS provides me an opportunity to build a multidisciplinary research institute. I learn to mentor early career and mid career researchers, and look after other colleagues as well as building the external partnerships and connections. There are completely different challenges. In fact, these challenges also teach me finding the solutions to make the Institute financially sustainable. The mentorship of ECRs and MCRs led to more than ten fellowships externally funded, and our strategic partnership developments with industry and other universities have resulted in several major research centres being funded, including the Australian IDEAL research hub. The establishment of IDEAL Hub further gave us a new credit as the leader in point of care diagnostics, which was strengthened by the Australian Department of Industry and its counterpart in China to fund the Joint Research Center in this area.

**Figure 4:**
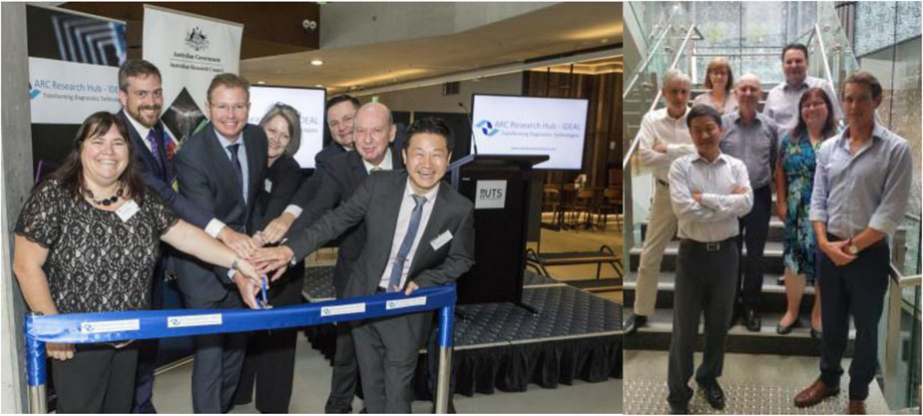
**An IDEAL way to connect with industry. In 2016, through the establishment of the IDEAL Hub, Professor Jin puts Australian biophotonics research into a world leading position, with strong industry partners. The ARC highlights: “A good researcher thinks about collaborations and takes action to develop them as they build their research career”**

**Q4: You talked about your success in attracting major research funding in Australia and China. We are also interested in learning how you could manage to publish more than 200 high-quality research papers, including more than 30 in Nature and its sister journals. Would you please share with us your experience and advice on securing research incomes and publishing the high-quality research outputs?**

The short answer is to align the research to the end-users’ needs. It becomes a lot of easier to sell the research significance, benefits and impacts to the funding agents, the editors and referees. I foster an environment that stimulates our young ECRs and postgraduate students to formulate their own big ideas, and encourage them to conduct high-risk and high-return projects. Most of our work has been designed to address the critical needs, and provide the potential solutions to the multidisciplinary research communities. This ensures the topic of each project not only very interesting in knowledge advancement, but also very trendy at the cutting edge of the emerging fields.

The second tip is to continuously build new instruments, though takes longer time to build, always return to us new capability, so that we could comprehensively investigate the science and suggest the new directions with sufficient depth. Majority of our high impact papers are based on the purpose-built instruments and instrumentation methods^2^, which makes our research unique, well-planned, innovative, and comprehensive.

I have a simple formula (why – what – how – and so what) for them to follow in their project planning, data presentation and publication preparations. “Why” your research is more important, and “why” the editors, the readers, and the reviewers should care about your research. “What” exactly you did, or what is exactly the idea? What’s fundamentally new, what’s fundamentally different from others’ doing? “How” actually you achieve this? How do you design your research very comprehensively, so as to make sure there are sufficient steps to take from where we are to where we want to be. Then “so what”? how the research will impact the community?

**Q5: You spent the past three years strengthening international collaborations, and established the UTS-SUSTech Joint Research Center for Biomedical Materials & Devices at Southern University of Science and Technology since 2019. What are your views on the Joint Research Center model for international collaboration?**

Biomedical research is a typical field requiring multidisciplinary skills and joint efforts from all the countries. As nobody could use a single technology to solve any single problem, you need a group of people to work together, and the research will benefit everyone in the world. I hope, through the scientific research, all the people around the world, all the nations, could reach a common understanding and recognition towards the mutual benefits.

From 2019, I took the new challenge again to explore the new model for international partnerships. I spent quite a lot of efforts in South Korea, Singapore, Germany, and China. The establishment of the JRC with SUSTech is a successful example. The JRC provides us with the scale we need to develop new technologies and prototype devices for tracking disease progression at the level of single cells. Bringing together the leading regional universities of technology, students spend 2 years in Shenzhen and 2 years in Sydney to gain the international experience, and researchers could develop sustainable model by jointly supervising PhD students and academic exchanges.

**Figure 5:**
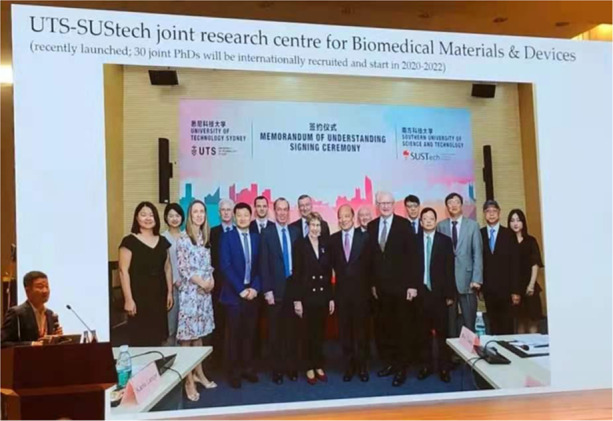
**Professor Jin accounced on an international conference the International biomedical partnership focuses on fine details. NSW Governor, the Hon Margaret Beazley AO QC. (middle of photo) witnessed MOU signing with SUSTech**

**Q6: As a world leader in photon-upconversion materials and technologies, your research breakthroughs have been published by the top-tier journals, including Nature, Nature Nanotechnology, Nature Photonics, Nature Methods, and Light: Science & Applications. Which one in particular you are most proud of? And how the series of advances been made? Where the field will go as the next?**

Welcome to the backyield of my “dots” collection! They began with Super Dots, the world-first highly doped upconversion nanocrystals, that are three orders of magnitude more sensitive than quantum dots. In our 2013 Nature Nanotechnology paper^3^, we showed that this limitation of concentration quenching can be overcome, so that we can significantly increase the brightness of single nanocrystal.

The dots that shine at different times were named Tau-Dots. In our 2014 Nature Photonics paper^4^, by controlling each Dot to ‘take a turn’ to shine, Tau-Dots could facilitate the high-throughput simultaneous detections of multiple target biological cells, subcellular components, and pathogen DNA molecules.

Then came Hyper Dots, in forming heterogeneously doped nanocrystals with the precise control in size, shape, surface, and composition placements (2016 Nature Communications^5^). We can build a single Dot into a nano machine.

We then unveiled a photon avalanche effect that amplifies stimulated emission in single Super Dots (2017 Nature^6^) that starts the new direction of upconversion super-resolution imaging.

And the dots for nanoscale temperature measurement were named Thermal Dots (2018 Nature Photonics^7^).

In our 2018 Light: Science & Application^8^ paper, we demonstrated the brightness of single crystal can be tracked by our naked eyes through a microscope, which provides a tool to study the intracellular transport of single molecules.

**Figure 6:**
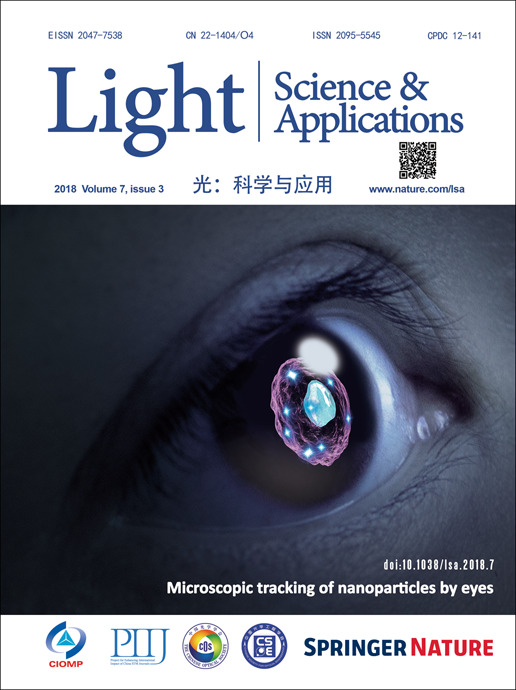
**LSA cover**

Most recently this “dots” series has been extended to nanomechanical force measurement in living cells (2021 Nature Nanotechnology^9^).

These nanocrystals can all be used for quantitative analysis of biochemical molecules. They can be transported to the point of interest, within a cell, and facilitate measurements of unprecedented sensitivity, down to the single-molecule level.

Infrared light can pass through biological tissue to illuminate the dots which then only emit visible and ultraviolet light, which is easily distinguishable from background light. This upconversion of the infrared light enables imaging deep into the body at high resolution and contrast.

Diagnostics in the future can become much faster and more specific using high-throughput simultaneous detection of multiple target cells, subcellular organelles, and pathogen molecules.

**Q7: To assist our response to the COVID-19 pandemic, your team has developed a rapid COVID-19 test. It only takes 15** **min to report the result, and the cost of each test is no more than AUD 25. Could you please tell us the principle of this technology? And how soon we could have the products?**

This is a very hot topic these days. The current PCR technology used in the market can biochemically amplify the single RNA molecule from the virus. After about 40 cycles, you got a trillion of the copies of oligo molecules, making this technology very sensitive, but it takes too long to have the result. It’s a laboratory-based test, not suitable for point of care testing.

**Figure 7:**
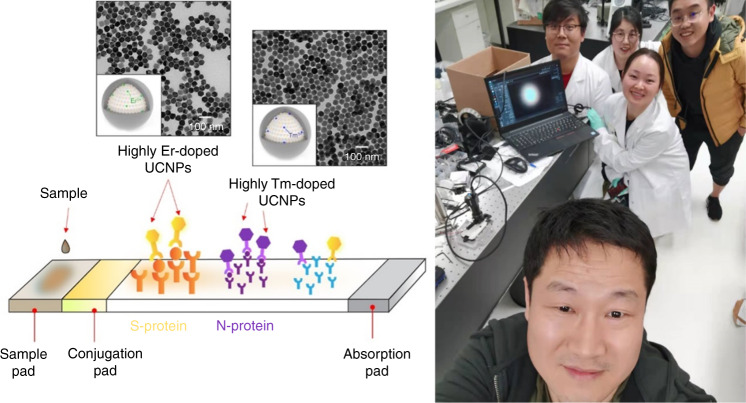
**Australian-made COVID-19 iStrip test returns results within minutes. Super Dot emits intense light in unexpected ways, known as upconversion, that provides ultra-sensitivity by converting infrared into visible light. The selfie photo of the COVID team, taken at the midnight of 31 July 2020, when the team achieved the first milestone of the project (photo on the right)**

Our strategy is to target the virus proteins and use Super Dots to optically amplify the signal. After lysing the virus by soap which takes a few seconds, each virus will release a few hundreds of protein fragments, including the spike antigen molecules from the surface. We then take advantage of Super Dots with thousands of emitters to optically amplify each molecule and significantly improve the detection sensitivity. The science is not new, as in our 2018 Analytical Chemistry^10^ paper, we first applied Super Dots in lateral flow assays and achieved a new LoD of 89 pg/mL for the detection of prostate-specific antigen proteins.

Since the pandemic, my team has optimized the same technology for the detection of virus antigen in asymptomatic people. The new test is more than two orders of magnitude more sensitive than the antigen tests now available on the market. The Perth-based Alcolizer Technology has licensed this invention from UTS and branded the strip and detector technologies as iStrip^TM^ and Virulizer^TM^. Hopefully our technology could contribute to the recovery of the pandemic, and to be used in the airport, shops, and nursing homes. As scientists, this makes us very excited, but the commercialization process will depend on the commercial decisions by our industry partner as well as the government regulatory process.

**Q8: Since 2012, you have participated in all the series of Light Conference and Light Young Scientist Forum. In 2018, you started the “Perspective” column for LSA, and contributed the first Light Online keynote talk in 2020, as well as judging for Light Doctoral Academic League in 2021. As the author, reviewer, editor, and regional manager of LSA and eLight, you witnessed the growth of our Light community. Would you like to comment about your experience with Light?**

My experience with LSA is always pleasant. With the strong supports by Changchun Institute of Optics, Fine Mechanics and Physics (CIOMP), Chinese Academy of Sciences (CAS), as well as Springer Nature, the excellent editorial management team has paved a great foundation for LSA. The success of LSA is beyond the profile of being a high impact journal. On each annual Light Conference and Young Scientist Forum, I enjoy the engagement opportunities, and observe the energy, the passion, and the enthusiasm there. The recent engagement channels, through Light Online, Light Doctorial Academic League, iCAN talks, and etc., further take onboard the broader international community and postgraduate students. Light sets a great example for publishing business to better engage with the research community with a strong focus on our students. It is a sustainable forum for our young researchers to show their work and to build research connections and networking opportunities. After nearly 10 years of rapid growth on the solid foundation, I have my confidence on the sustainability of Light community. I am very proud of being part of it.

My roles as the manager in Sydney Office, and the editor for the “Perspective” column gave me the opportunity to stay close with our world-class researchers, and to learn the latest developments at the leading edge of the emerging fields of photonics. I often spend long emails and phone calls, as well as the face to face discussions, with our leading researchers to identify the best Perspective topics. I would like to take this opportunity to encourage our authors to send me emails and arrange time to discuss the emerging directions and the future perspectives of optics and photonics.

**Figure 8:**
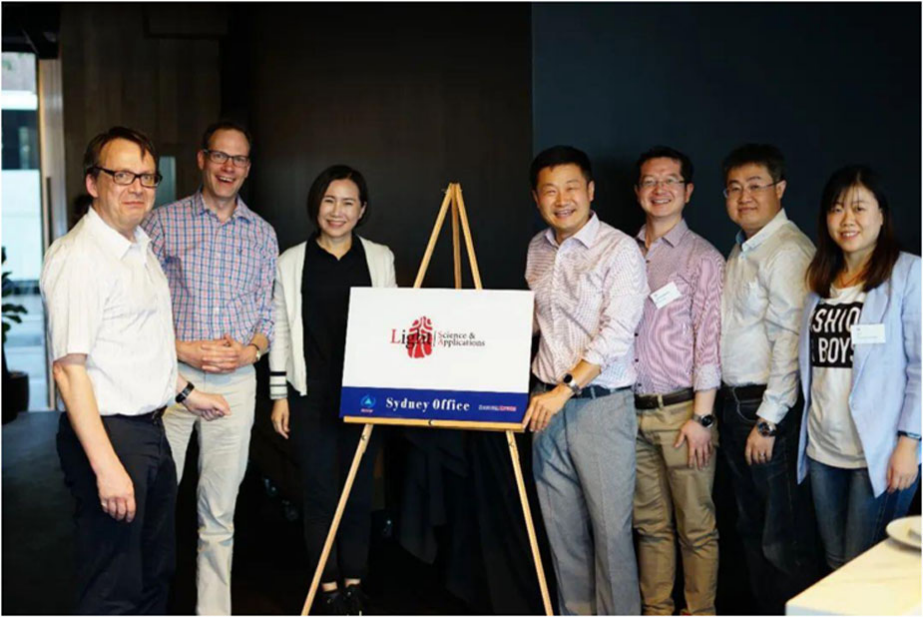
**Opening ceremony of LSA Sydney office in 2018**

**Q9: As the principal supervisor of more than 30 PhD students, including 19 completions to date, could you please tell our future students how you select students and how to cultivate them to become high-quality researchers?**

I’m very proud of my past and current students, and sincerely acknowledge their commitments and contributions to our success as a team. I had 8 PhD students at Macquarie, another 24 students at UTS, and now in the process of recruiting and supervising another 30 or so students at SUSTech and UTS, which is my goal in supervision in the next five years. I would like to take this opportunity to welcome the potential candidates from disciplines, such as photonics, analytical methods, cell biology, data analytics, and device engineering, to join our ever-growing IBMD institute.

**Figure 9:**
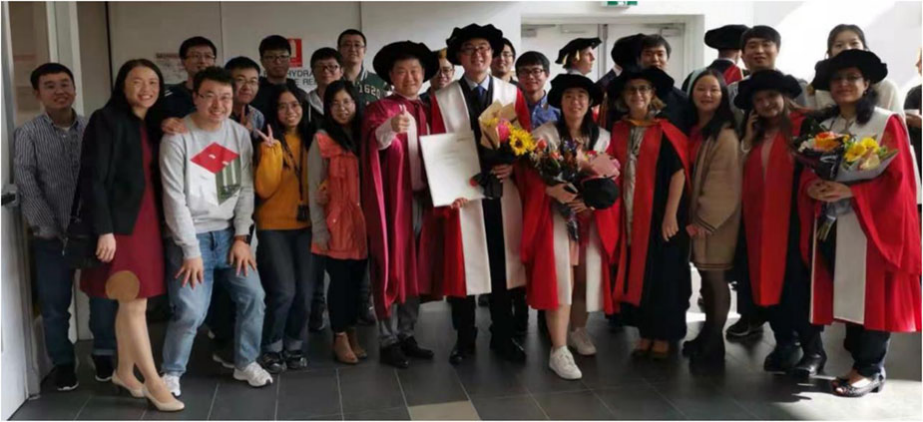
**Graduation ceremony**

When I select a student, I expect them with the proper research training, being optimistic with the right attitude, and being persistent with a strong desire for science research.

At the beginning of their PhD studies, I encourage our students to challenge the big science questions, and to conduct high-risk and high-return projects. To minimize the risk, I formulate three principles to guide them on how to choose the right research topic:

the student should feel very interested in pursuing the new knowledge around the topic they choose;

the student should have the right skills to start the research, while seeking to learn the new skills;

the topic should be within the scope of IBMD environment, and overlap to a certain degree with their peers’ projects, so that they could turn to a peer for discussions whenever they encounter the issues in the lab. It is also good for them to collaborate with each other.

Through the project, I will teach my students to equip themselves the three types of skills:

The professional skills include both the experimental and analytical aspects.

The communication skills include the writing and presentation skills, the collaboration and networking skills and skills to work on the project both independently and as a team. This is most essential to our multidisciplinary research.

I will then show my students how to plan, how to manage the time and resources, and how to develop the strategic thinking skills. Many of my students, after PhD completion, have learned how to do, how to communicate, and how to think and plan.

**Light correspondent**

*Dr. Ying Zhang is the vice director of Light Publishing Group at Changchun Institute of Optics, Fine Mechanics and Physics (CIOMP), Chinese Academy of Sciences (CAS). He has been a visiting scholar at the Institute of Optics, University of Rochester during 2017–2018. He currently serves as the executive editor-in-chief of Chinese Journal of Liquid Crystals and Displays as well as an academic editor of Light: Science & Applications. He won the first prize and outstanding contribution award of project supported by STM Journal Society, CAS. He has hosted and participated in more than 10 provincial and ministerial projects of China. He has published over 40 SCI/EI and management papers. He also has presented over 30 invited talks and has been interviewed by China****’****s mainstream media such as the Xinhua News Agency. He participated in organizing and editing Handbook of Laser Technology and Applications (2nd Ed.) published by CRC, Taylor & Francis Group, as well as collection “Publishing Ethics of STM Periodicals” organized by China Association for Science and Technology*.

**Figure:**